# Ought Members of the Active Medical Staffs to Sit upon Hospital Boards?

**Published:** 1890-03-01

**Authors:** 


### Passing Topics.

### OUGHT MEMBERS OF THE ACTIVE MEDICAL STAFFS TO SIT UPON HOSPITAL BOARDS?—II.

Something would be gained were it possible to regard the claim of the medical officers, as illustrated at the German Hospital, not merely as a subject of controversy between them and the committee in regard to which one party is to achieve a victory over the other, but rather as a question for frank and friendly debate, in which nothing shall be said to cause needless irritation, but every effort shall be made to compass a conclusion honourable and satisfactory to both sides.

We think the claimants for representation start with a misapprehension, inasmuch as they assume that they are "honorary" officers. There can be no more misleading term, and this must be borne in mind in order to dispose of the suggestion that the hospitals are under so great obligation to their staffs as to be compelled in common gratitude to grant them uncommon rights and privileges. The matter should be discussed on its merits. Both definite duties and definite advantages attach to the position of a hospital physician or surgeon, and it is a straining of language to call any such appointment an honorary one. What is meant is that the visiting staff do not receive remuneration in hard cash for services rendered. None the less, the advantages are so great and tangible that hospital appointments are sought after with the utmost eagerness by the most promising members of the profession, and are held with tenacity by men of the very highest attainments. They constitute the only real passports to eminence if not fortune, and so all-important is connection with a hospital known to be that, failing ability to obtain a staff appointment upon an established hospital, ambitious practitioners take to "founding" hospitals in order to create positions for themselves.

To the purely medical mind, nothing is more self-evident than the inadaptability of lay management to the aims and requirements of a medical institution; and were it not that the financial necessities render lay co-operation indispensable, the more ardent exponents of professional policy would hardly rest content with "representation." Yet experienced physicians would agree that there could be no greater misfortune for the institutions or their beneficiares and their staffs than that they should become mere scientific laboratories. It is as pertinent to ask how long the patients would be found willing to supply in their persons the raw material for manipulation as to inquire whether the benevolent would still continue to support institutions administered chiefly in the interests of science. It is already something more than a surmise that a scientific reputation hinders as much as it helps a hospital. Many of the very best friends hospitals possess refuse to recognise them as anything but homes of relief for the suffering poor, and wince at too prominent a suggestion of their scientific value. Whatever is calculated to impair the feeling of confidence in the philanthropic and religious character of the work ought to be carefully avoided, and it is well known that professional members of a committee are apt to acquire a power altogether out of proportion to their numbers.

It is no doubt as true as it is unfortunate that Hospital Committees are not always distinguished by their efficiency. Members may be neither regular in attendance, nor, if present, are they invariably capable and well informed. In the last case they cannot fail to prove unsympathetic in the treatment of matters about which, perhaps, the staff rightly entertain strong feelings.

But, whatever the legitimate requirements of the staff, we think they may be adequately met by the medical committee, which usually has an existence of some sort, but is not always prompt to make use of its opportunities. A medical committee, working side by side with the governing body, and making its recommendations, must be able to procure acquiescence in every rightful demand, while leaving the authority of the latter unbroken, and avoiding the danger of discord and weakness, which, as experience at many a hospital has shown, is only too readily generated by the presence of elements incapable of fusion with the rest of the body, and constituting a veritable *imperium in imperio*.

This appears to be the view entertained by the authorities of the most important hospitals, and it becomes the more obviously reasonable when we reflect that the physicians and surgeons of the institution are among its executive officers. Whatever the custom at many, chiefly of the smaller, hospitals, the representation of the staff upon the governing body, appears wrong in principle, and any wider application of an arrangement those best able to judge have found faulty might prove a misfortune. Agricola.

